# Supplementary material for: Pyruvate Dehydrogenase Kinase 1 inhibition mediated oxidative phosphorylation enhancement in cartilage promotes osteoarthritis progression
Source: BMC Musculoskelet Disord. 2023 Jul 20;24:597. doi: 10.1186/s12891-023-06585-6 (PMC10357736; doi:10.1186/s12891-023-06585-6)
Supplement: Supplementary file 1 — Additional file 1. [file 12891_2023_6585_MOESM1_ESM.zip › KEGG.Gsea.1653622432464/KEGG_RNA_DEGRADATION.html]

Details for gene set KEGG\_RNA\_DEGRADATION[GSEA]

|  || Dataset | 55457\_collapsed\_to\_symbols.cls.cls #OA\_versus\_normal.cls.cls #OA\_versus\_normal\_repos |
| Phenotype | cls.cls#OA\_versus\_normal\_repos |
| Upregulated in class | OA |
| GeneSet | KEGG\_RNA\_DEGRADATION |
| Enrichment Score (ES) | 0.22593914 |
| Normalized Enrichment Score (NES) | 0.9129738 |
| Nominal p-value | 0.6175 |
| FDR q-value | 0.7878837 |
| FWER p-Value | 1.0 |
Table: GSEA Results Summary

  

Fig 1: Enrichment plot: KEGG\_RNA\_DEGRADATION      
 Profile of the Running ES Score & Positions of GeneSet Members on the Rank Ordered List

  

| SYMBOL | TITLE | RANK IN GENE LIST | RANK METRIC SCORE | RUNNING ES | CORE ENRICHMENT || 1 | LSM4 | "LSM4 homolog, U6 small nuclear RNA and mRNA degradation associated [Source:HGNC Symbol;Acc:HGNC:17259]" | 174 | 0.837 | 0.0444 | Yes |
| 2 | CNOT4 | CCR4-NOT transcription complex subunit 4 [Source:HGNC Symbol;Acc:HGNC:7880] | 347 | 0.724 | 0.0812 | Yes |
| 3 | LSM3 | "LSM3 homolog, U6 small nuclear RNA and mRNA degradation associated [Source:HGNC Symbol;Acc:HGNC:17874]" | 377 | 0.716 | 0.1285 | Yes |
| 4 | WDR61 | WD repeat domain 61 [Source:HGNC Symbol;Acc:HGNC:30300] | 711 | 0.590 | 0.1435 | Yes |
| 5 | LSM1 | "LSM1 homolog, mRNA degradation associated [Source:HGNC Symbol;Acc:HGNC:20472]" | 1470 | 0.433 | 0.1145 | Yes |
| 6 | CNOT7 | CCR4-NOT transcription complex subunit 7 [Source:HGNC Symbol;Acc:HGNC:14101] | 1676 | 0.402 | 0.1263 | Yes |
| 7 | DDX6 | DEAD-box helicase 6 [Source:HGNC Symbol;Acc:HGNC:2747] | 1687 | 0.401 | 0.1533 | Yes |
| 8 | C1D | C1D nuclear receptor corepressor [Source:HGNC Symbol;Acc:HGNC:29911] | 1848 | 0.379 | 0.1671 | Yes |
| 9 | EXOSC7 | exosome component 7 [Source:HGNC Symbol;Acc:HGNC:28112] | 1969 | 0.364 | 0.1830 | Yes |
| 10 | LSM5 | "LSM5 homolog, U6 small nuclear RNA and mRNA degradation associated [Source:HGNC Symbol;Acc:HGNC:17162]" | 2127 | 0.347 | 0.1948 | Yes |
| 11 | MTREX | Mtr4 exosome RNA helicase [Source:HGNC Symbol;Acc:HGNC:18734] | 2138 | 0.346 | 0.2179 | Yes |
| 12 | DCPS | "decapping enzyme, scavenger [Source:HGNC Symbol;Acc:HGNC:29812]" | 2570 | 0.295 | 0.2048 | Yes |
| 13 | DCP2 | decapping mRNA 2 [Source:HGNC Symbol;Acc:HGNC:24452] | 2659 | 0.284 | 0.2176 | Yes |
| 14 | TTC37 | tetratricopeptide repeat domain 37 [Source:HGNC Symbol;Acc:HGNC:23639] | 2875 | 0.263 | 0.2191 | Yes |
| 15 | PARN | poly(A)-specific ribonuclease [Source:HGNC Symbol;Acc:HGNC:8609] | 3100 | 0.240 | 0.2183 | Yes |
| 16 | ENO3 | enolase 3 [Source:HGNC Symbol;Acc:HGNC:3354] | 3207 | 0.230 | 0.2259 | Yes |
| 17 | LSM7 | "LSM7 homolog, U6 small nuclear RNA and mRNA degradation associated [Source:HGNC Symbol;Acc:HGNC:20470]" | 3627 | 0.191 | 0.2066 | No |
| 18 | CNOT1 | CCR4-NOT transcription complex subunit 1 [Source:HGNC Symbol;Acc:HGNC:7877] | 3842 | 0.172 | 0.2018 | No |
| 19 | LSM6 | "LSM6 homolog, U6 small nuclear RNA and mRNA degradation associated [Source:HGNC Symbol;Acc:HGNC:17017]" | 4019 | 0.154 | 0.1988 | No |
| 20 | DIS3 | "DIS3 homolog, exosome endoribonuclease and 3'-5' exoribonuclease [Source:HGNC Symbol;Acc:HGNC:20604]" | 4187 | 0.139 | 0.1955 | No |
| 21 | EXOSC5 | exosome component 5 [Source:HGNC Symbol;Acc:HGNC:24662] | 4295 | 0.128 | 0.1960 | No |
| 22 | CNOT8 | CCR4-NOT transcription complex subunit 8 [Source:HGNC Symbol;Acc:HGNC:9207] | 4581 | 0.106 | 0.1812 | No |
| 23 | LSM2 | "LSM2 homolog, U6 small nuclear RNA and mRNA degradation associated [Source:HGNC Symbol;Acc:HGNC:13940]" | 4641 | 0.101 | 0.1836 | No |
| 24 | MPHOSPH6 | M-phase phosphoprotein 6 [Source:HGNC Symbol;Acc:HGNC:7214] | 5077 | 0.067 | 0.1544 | No |
| 25 | ENO2 | enolase 2 [Source:HGNC Symbol;Acc:HGNC:3353] | 5572 | 0.026 | 0.1178 | No |
| 26 | EXOSC4 | exosome component 4 [Source:HGNC Symbol;Acc:HGNC:18189] | 5693 | 0.016 | 0.1096 | No |
| 27 | CNOT2 | CCR4-NOT transcription complex subunit 2 [Source:HGNC Symbol;Acc:HGNC:7878] | 5740 | 0.012 | 0.1068 | No |
| 28 | CNOT3 | CCR4-NOT transcription complex subunit 3 [Source:HGNC Symbol;Acc:HGNC:7879] | 6183 | -0.019 | 0.0738 | No |
| 29 | LSM8 | "LSM8 homolog, U6 small nuclear RNA associated [Source:HGNC Symbol;Acc:HGNC:20471]" | 6828 | -0.065 | 0.0282 | No |
| 30 | PAPOLG | poly(A) polymerase gamma [Source:HGNC Symbol;Acc:HGNC:14982] | 7268 | -0.100 | 0.0009 | No |
| 31 | DCP1A | decapping mRNA 1A [Source:HGNC Symbol;Acc:HGNC:18714] | 7525 | -0.121 | -0.0106 | No |
| 32 | PAPOLB | poly(A) polymerase beta [Source:HGNC Symbol;Acc:HGNC:15970] | 7906 | -0.148 | -0.0299 | No |
| 33 | SKIV2L | Ski2 like RNA helicase [Source:HGNC Symbol;Acc:HGNC:10898] | 8395 | -0.185 | -0.0551 | No |
| 34 | EXOSC10 | exosome component 10 [Source:HGNC Symbol;Acc:HGNC:9138] | 8404 | -0.186 | -0.0428 | No |
| 35 | EXOSC8 | exosome component 8 [Source:HGNC Symbol;Acc:HGNC:17035] | 8554 | -0.198 | -0.0407 | No |
| 36 | HSPA9 | heat shock protein family A (Hsp70) member 9 [Source:HGNC Symbol;Acc:HGNC:5244] | 9173 | -0.246 | -0.0718 | No |
| 37 | ENO1 | enolase 1 [Source:HGNC Symbol;Acc:HGNC:3350] | 9606 | -0.284 | -0.0858 | No |
| 38 | PAPOLA | poly(A) polymerase alpha [Source:HGNC Symbol;Acc:HGNC:14981] | 9786 | -0.300 | -0.0789 | No |
| 39 | TENT4A | terminal nucleotidyltransferase 4A [Source:HGNC Symbol;Acc:HGNC:16705] | 10003 | -0.322 | -0.0734 | No |
| 40 | EXOSC9 | exosome component 9 [Source:HGNC Symbol;Acc:HGNC:9137] | 10606 | -0.384 | -0.0937 | No |
| 41 | EDC4 | enhancer of mRNA decapping 4 [Source:HGNC Symbol;Acc:HGNC:17157] | 10686 | -0.391 | -0.0727 | No |
| 42 | CNOT6 | CCR4-NOT transcription complex subunit 6 [Source:HGNC Symbol;Acc:HGNC:14099] | 11212 | -0.459 | -0.0818 | No |
| 43 | HSPD1 | heat shock protein family D (Hsp60) member 1 [Source:HGNC Symbol;Acc:HGNC:5261] | 11427 | -0.492 | -0.0644 | No |
| 44 | EXOSC2 | exosome component 2 [Source:HGNC Symbol;Acc:HGNC:17097] | 12100 | -0.620 | -0.0737 | No |
| 45 | CNOT9 | CCR4-NOT transcription complex subunit 9 [Source:HGNC Symbol;Acc:HGNC:10445] | 12668 | -0.839 | -0.0598 | No |
| 46 | EDC3 | enhancer of mRNA decapping 3 [Source:HGNC Symbol;Acc:HGNC:26114] | 12868 | -1.123 | 0.0025 | No |
Table: GSEA details [plain text format]

  

Fig 2: KEGG\_RNA\_DEGRADATION      
 Blue-Pink O' Gram in the Space of the Analyzed GeneSet

  

Fig 3: KEGG\_RNA\_DEGRADATION: Random ES distribution      
 Gene set null distribution of ES for **KEGG\_RNA\_DEGRADATION**
